# High-Energy Long-Lived Emitting Mixed Excitons in Homopolymeric Adenine-Thymine DNA Duplexes

**DOI:** 10.3390/molecules27113558

**Published:** 2022-05-31

**Authors:** Ignacio Vayá, Thomas Gustavsson, Dimitra Markovitsi

**Affiliations:** 1 Departamento de Química, Instituto de Tecnología Química UPV-CSIC, Universitat Politècnica de València, 46022 Valencia, Spain; igvapre@qim.upv.es; 2 Université Paris-Saclay, CEA, CNRS, LIDYL, 91191 Gif-sur-Yvette, France; thomas.gustavsson@cea.fr; 3 Université Paris-Saclay, CNRS, Institut de Chimie Physique, UMR8000, 91405 Orsay, France

**Keywords:** DNA, intrinsic fluorescence, time-resolved fluorescence spectroscopy, excitons, hypochromism

## Abstract

The publication deals with polymeric pA●pT and oligomeric A_20_●T_20_ DNA duplexes whose fluorescence is studied by time-correlated single photon counting. It is shown that their emission on the nanosecond timescale is largely dominated by high-energy components peaking at a wavelength shorter than 305 nm. Because of their anisotropy (0.02) and their sensitivity to base stacking, modulated by the duplex size and the ionic strength of the solution, these components are attributed to mixed ππ*/charge transfer excitons. As high-energy long-lived excited states may be responsible for photochemical reactions, their identification via theoretical studies is an important challenge.

## 1. Introduction

A series of articles published since the beginning of this century [1,2,3,4,5] showed that time-resolved fluorescence spectroscopy (TRFS) is a very sensitive tool for studying excited state relaxation in DNA duplexes, despite their extremely low fluorescence quantum yields ϕ, which is of the order of 10^−4^. Compared to transient absorption spectroscopy, probing strongly populated excited states [4,6,7,8,9], fluorescence spectroscopy is capable of detecting emission related to minor populations, that evolve along different deactivation routes. Thus, for all the studied DNA duplexes, TRFS has revealed the existence of long-lived excited states decaying on the nanosecond timescale, while those monitored by transient absorption decay on the sub-ns timescale.

As highlighted recently [5], the long-lived DNA fluorescence may be classified in two families. On the one hand, those appearing in the visible spectral domain and peaking around 420–450 nm; these were already reported more than forty years ago [10,11,12,13,14,15,16] and attributed to excimers. Quantum chemistry calculations evidenced the strong charge transfer (CT) character of the low-energy states [17,18,19,20,21], in agreement with the negative fluorescence anisotropy *r* determined for DNA multimers at these wavelengths (see Figure 4c in reference [5]). More recently, fluorescence peaking in the UV and characterized by slightly positive *r* values (0.02–0.03) was discovered [22,23,24,25]. The most striking example is provided by double-stranded structures with an alternating guanine-cytosine sequence (GC)_n_●(GC)_n_, whose steady-state fluorescence spectrum is largely dominated by an emission band peaking at around 305 nm [22,23,24]. Similar features, albeit less visible in the steady-state fluorescence spectra due to overlapping with emission from other types of excited states, were also found for duplexes with an alternating adenine-thymine sequence (AT)_n_●(AT)_n_ and also in genomic DNA [25]. These intriguing long-lived emitters were denoted **H**igh-energy **E**mitting **L**ong-lived **M**ixed excitons, or more simply HELM excitons. Quantum chemistry studies proposed their assignment to mixed ππ*/CT exciton states, extended over both strands and characterized by a weak intrastrand purine-to-pyrimidine charge transfer [24,25].

**Figure 1:**
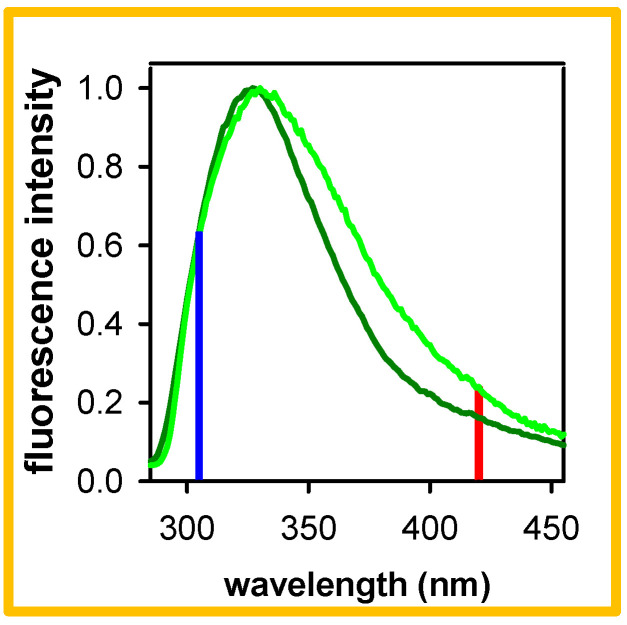
Steady-state fluorescence spectra recorded for pA●pT (dark green) and A_20_●T_20_ (light green) following excitation at 267 nm [30]; their intensity was normalized at the maximum; vertical blue and red lines indicate, respectively, 305 and 420 nm, where fluorescence decays and fluorescence anisotropy traces are shown herein.

In the beginning of this year, Ma, Kwok et al. reported dual long-lived fluorescence for RNA duplexes composed by homopolymeric adenine-uracil sequences [26]. A “high-energy” component, peaking at 324 nm and characterized by a lifetime of 1.7 ns, was attributed to “electronically neutral excitonic states”, because their anisotropy (0.15) is significantly higher compared to that of HELM excitons. The occurrence of high-energy long-lived fluorescence in these RNA duplexes appeared to contrast with the absence of such emission from their DNA analogs, composed of homopolymeric adenine-thymine sequences. As a matter of fact, several years ago, the fluorescence of such DNA duplexes was studied over six orders of magnitude of time by two authors of the present work [1,27,28,29,30]. Nevertheless, those studies had not at all explored the high-energy part of the fluorescence spectrum, which does not display any particular feature (Figure 1). An additional reason is that fluorescence measurements are more delicate at such short wavelengths. Since then, however, experimental protocols were improved, and thus we decided to investigate precisely this aspect, which was neglected in the past.

Here, we examine polymeric DNA duplexes with ~1000 base-pairs, and oligomeric ones with twenty base pairs, noted, respectively, pA●pT and A_20_●T_20_. The structure of such duplexes has been extensively characterized by X-ray and CD measurements, which revealed that they adopt a B-type of double helix with some particularities (e.g., narrowing of the minor grove) [31,32,33]. For most of the experiments, they were dissolved in saline phosphate buffer (0.03 mol●L^−1^ Na_2_HPO_4_, 0.03 mol●L^−1^ NaH_2_PO_4_, 0.48 mol●L^−1^ NaCl), to which we refer as “standard buffer”. We report time-resolved fluorescence spectra, fluorescence decays and fluorescence anisotropies obtained by time-correlated single photon counting (TCSPC) with an excitation wavelength of either 267 nm or 285 nm. The results presented and discussed hereafter clearly show the existence of fluorescence components stemming from HELM excitons, despite the fact that their fingerprint is not observable in the steady-state emission spectra (Figure 1).

## 2. Results

**Figure 2:**
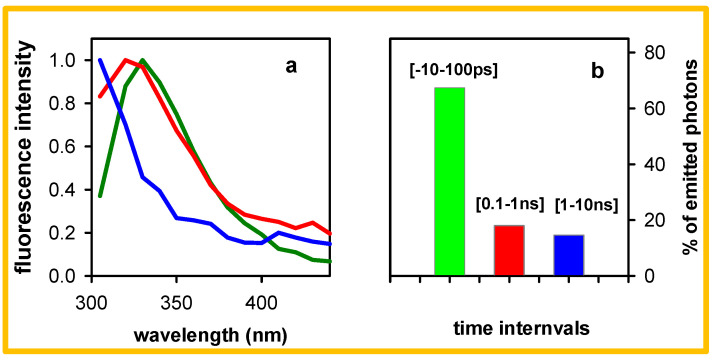
Spectral and temporal distribution of the photons emitted by of pA●pT following 267 nm excitation: (**a**) spectra (305–440 nm) integrated over the time-windows [−50–50 ps] (green), [0.1–1 ns] (red) and [1–10 ns] (blue) and normalized at their maximum; (**b**) percentage of the total number of photons emitted at successive time intervals on the 305–440 nm range.

Time-resolved spectra determined for the polymeric duplex at selected time-windows are shown in Figure 2a. They were reconstructed from a series of fluorescence decays by integrating the photons emitted at each wavelength over the considered time-window; subsequently, they were corrected for the spectral response of the detection system and normalized at their maximum to better reveal spectral changes. Raw spectra are shown in the Supplementary Materials (Figure S1). The spectrum of the photons emitted from −50 to 50 ps (time-width entirely within the apparatus function) peaks at 330 nm. Between 0.1 and 1 ns, the peak shifted to 320 nm, while a weak intensity shoulder appeared around 420 nm. On the ns timescale, the emission band shifts further to the blue, the maximum likely located at wavelengths shorter than 305 nm; unfortunately, this region is unexploitable because of the Raman line of water. In parallel, the relative intensity of the shoulder located in the visible further decreases; the photons emitted in the visible range (410–440 nm) represent now only 20% of the photons emitted in the UV (305–400 nm). In fact, this percentage should be even lower taking into account that the UV part of the spectrum is truncated. The percentage of the total number of photons emitted at three successive time intervals over the ensemble of the probed wavelengths is illustrated in Figure 2b. They were obtained by integrating the numbers of emitted photons at all the probed wavelengths over the considered time-window and, subsequently, divided by the overall number of photons. The photons emitted between 1 and 10 ns represent 15% of the total and ~12% correspond to the UV band.

**Figure 3:**
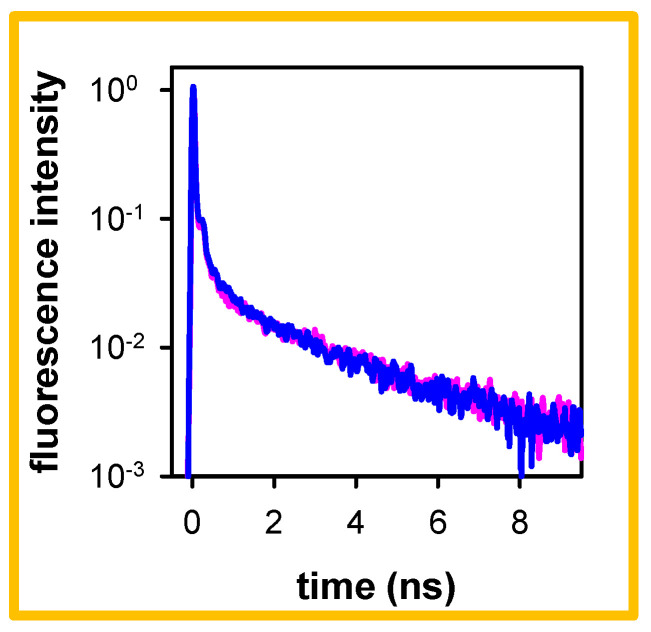
Normalized fluorescence decays recorded at 305 nm for pA●pT in the standard buffer (blue) and in KCl solution with the same ionic strength (pink); excitation wavelength: 267 nm.

At this stage, it is important to check if the high-energy long-lived fluorescence band, presented in Figure 2a, is not related to impurities of the salts used for the preparation of the buffer. Even if the purity of the used chemicals is higher than 99.99%, we cannot rule out such a possibility, given that we are dealing with ϕ values of the order of 10^−4^ and the buffer concentration is much higher than the DNA concentration (~10^−4^ mol●L^−1^ per base). Therefore, we replaced our standard buffer by a KCl aqueous solution with the same ionic strength and recorded the fluorescence decay at 305 nm. The latter is compared in Figure 3 with the corresponding decay obtained for the polymeric duplex in the standard buffer, used to construct the time-resolved spectra. The observation that the two decays are practically identical allows us to preclude that the high-energy fluorescence stems from buffer impurities. It is also worth noticing that the water used for the preparation of the solutions does not exhibit any detectable fluorescence.

**Figure 4:**
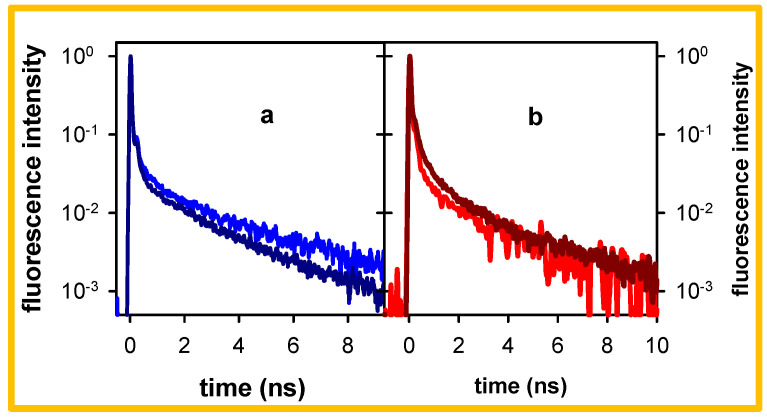
Normalized fluorescence decays recorded for pA●pT in standard buffer at 305 nm (**a**) and 420 nm (**b**) following excitation at 267 nm (blue, red) and 285 nm (dark blue, dark red).

The time-resolved spectra and the decays presented so far (Figure 2 and Figure 3) were obtained following excitation at 267 nm. As reported in reference [29], the steady-state fluorescence spectra remain the same when the excitation wavelength varies between 245 and 285 nm. Here, we found that when the excitation wavelength shifted from 267 nm to 285 nm, the 305 nm decay obtained for pA●pT becomes somewhat faster (Figure 4a). Small changes also appear for the 420 nm decay, representative of the low-energy fluorescence (Figure 4b). However, the percentage of photons emitted on the ns timescale remains more important at 305 nm compared to 330 nm (Table 1), close to the maximum of the steady state fluorescence spectrum (Figure 1).

The decays presented in Figure 4 are characterized by strongly non-exponential patterns. As a matter of fact, previous studies on pA●pT reported that its emission, probed by combining fluorescence upconversion and TCSPC measurements, starts decaying on the fs timescale [27,29] and at least five time constants are needed to fit the entire decays recorded at 330, 380 and 420 nm with 267 nm excitation [29]. However, the 305 nm decays obtained here can be approximated (but not perfectly described, see Figure S2) by single exponential functions when the fits are limited on the 1.5–10.5 ns range. We use these fits for phenomenological comparison between the data. The time constants determined in this way are 2.6 and 2.2 ns, respectively, for excitation at 267 and 285 nm (Table 2). In contrast, the 420 nm signals deviate more strongly from mono-exponentiality, even on the ns time-domain.

In order to quantify the relative importance *R* of the long-lived fluorescence at 305 nm, in respect to that at 420 nm, which for a long time have been considered as the only source of ns emission, we proceeded as follows. First, we determined for each decay the percentage of photons emitted on the ns timescale. Then, we multiplied this ratio by the relative intensities of the steady-state fluorescence spectrum at these wavelengths (Figure 1; equal to 3.9 for pA●pT) and determined *R* from Equation (1):

(1) $$R=\frac{\int_{1\;ns}^{\infty }I^{305}\left(t\right)dt/I_{ss}^{305}}{\int_{1\;ns}^{\infty }I^{420}\left(t\right)dt/I_{ss}^{420}}$$

The *R* values corresponding to both excitation wavelengths (Table 2) reveal the predominance of the high-energy long-lived fluorescence, which seems to be more important for the shorter excitation wavelength (5.6 vs. 3.6).

We now examine the comparison between oligomeric and polymeric duplexes. The size effect on the fluorescence dynamics was examined for excitation at 285 nm. As in the case of the polymer, the fluorescence decays of the oligomer contain long-lived components both at 305 nm and 420 nm. The percentage of photons emitted at these wavelengths by A_20_●T_20_ after 1 ns correspond, respectively, to 51% and 36%, vs. 27% and 28% for pA●pT (see also Table 1 for comparison between the 305 and 330 nm decays). Moreover, the relative importance of the long-lived high-energy emission *R*, determined according to Equation (1), is 3.7, a value similar to that found for pA●pT (Table 2). Additionally, for the smaller duplex, the 420 nm decay strongly deviates from mono-exponential behavior on the ns timescale. In contrast, the decay at 305 nm can be described by a mono-exponential function with a time constant of 2.7 ns (Figure S2), which is slightly longer than that found for the polymer under the same conditions (Table 2).

**Figure 5:**
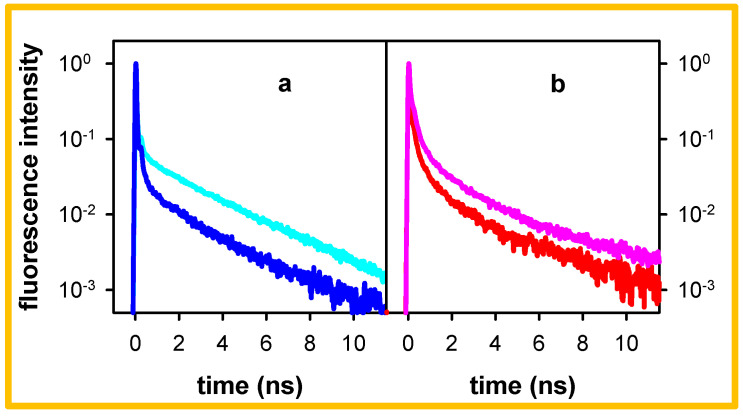
Comparison of the normalized fluorescence decays recorded for A_20_●T_20_ (cyan and pink) and pA●pT (blue and red) in standard buffer at 305 nm (**a**) and 420 nm (**b**); excitation wavelength: 285 nm.

**Figure 6:**
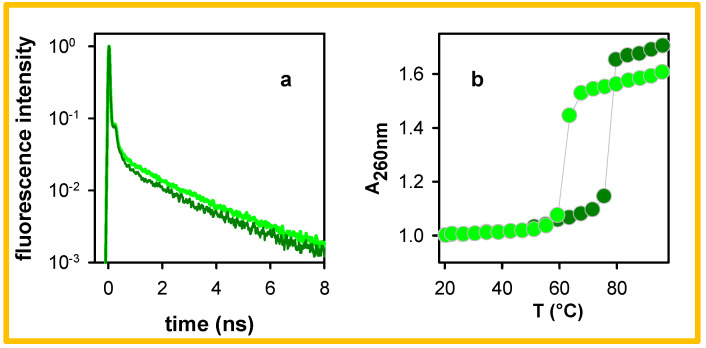
Normalized fluorescence decays at 305 nm (**a**) and melting curves at 260 nm (**b**) recorded for pA●pT in the standard buffer in its nominal concentration (dark green) and diluted by a factor six (light green); excitation wavelength: 285 nm.

A difference between the decays of the oligomer and the polymer shown in Figure 5 concerns the amplitude of the long-lived components: it is clearly higher for the shorter system at both 305 and 420 nm. A similar trend to that observed upon reducing the duplex size is also found when the polymer is dissolved in a buffer with a lower ionic strength. This is shown in Figure 6a, where the 305 nm decays of pA●pT in the standard buffer and in the buffer diluted by a factor of six are compared. Following such dilution of the buffer, the percentage of photons emitted at 305 nm after 1 ns increases from 27% to 31% (see also Table 1).

**Figure 7:**
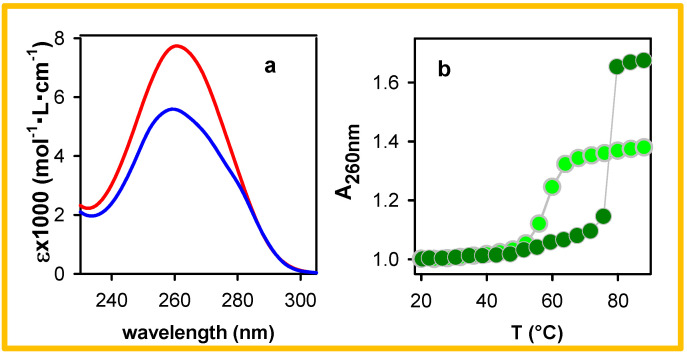
(**a**) Steady state absorption of A_20_●T_20_ at 20 °C (blue) and 92 °C (red); ε values (per base) were taken from reference [34]. (**b**) Melting curves determined at 260 nm for A_20_●T_20_ (light green) and pA●pT (dark green).

The increase in the amplitude of the long-lived components occurring upon decreasing the duplex size (Figure 5a) or reducing the ionic strength of the aqueous solvent (Figure 6a) can be correlated with changes in their absorption spectra observed upon melting, as shown in Figure 7a in the case of A_20_●T_20_. The variation of the absorbance at 260 nm is widely used to obtain the melting curves of duplexes. From the melting curves in Figure 6b and Figure 7b, it appears that the increase in the absorbance determined at 96 °C is larger for pA●pT (70%) compared to A_20_●T_20_ (40%), while that of pA●pT in the diluted buffer is reduced to 60%. Inversely, the hypochromism of the polymer is higher than that of the oligomer and it increases for the polymer when it is dissolved in a buffer with higher ionic strength.

**Figure 8:**
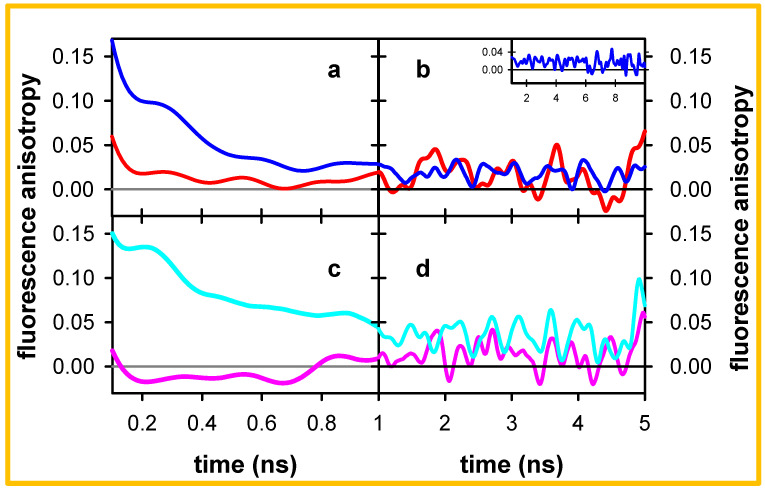
Fluorescence anisotropy traces determined for pA●pT (**a**,**b**) and A_20_●T_20_ (**c**,**d**) in standard buffer at 305 nm (blue, cyan) and 420 nm (red, pink) on the sub-ns (**a**,**c**) and ns timescales (**b**,**d**); excitation wavelength: 285 nm. Note that the time axis starts at 0.1 ns.

Finally, we examine the behavior of the fluorescence anisotropy at 305 and 420 nm for the polymeric and the oligomeric duplexes (Figure 8). For both systems, *r* at 305 nm has a value of ~0.15 at 0.1 ns and drops rapidly on a sub-ns timescale. Subsequently, *r* acquires a constant value of ~0.02 for the polymer and twice as high (~0.04) for the oligomer. In contrast, at 420 nm, the two systems have practically identical *r* values on the ns timescale (~0.02). However, on the shorter timescale, where *r* has lower values than at 305 nm for both systems, we remark a significant difference: the *r* determined for the oligomer between 0.2 and 0.8 ns is slightly negative (−0.01), while for the polymer *r* remains slightly positive (0.01).

## 3. Discussion

The results presented in Section 2 reveal that the fluorescence of both pA●pT and A_20_●T_20_ contain components emitting in the blue part of the fluorescence spectrum (Figure 1) and decaying with lifetimes of 2.2–2.7 ns (Table 1). This high-energy long-lived fluorescence is not related to any impurities, neither in the salts present in the aqueous solution (Figure 3) nor in the DNA samples themselves. In this respect, we note that the protocol followed for the preparation of the DNA single strands A_20_ and T_20_ (solid state synthesis), used to form the oligomeric duplex by annealing, differs from the biochemical synthesis of the double-stranded structure pA●pT. Yet, both systems exhibit high-energy long-lived emission.

Before further discussing the high-energy long-lived excited states of the examined duplexes, we briefly recall the main findings regarding their excited states, reported in the literature. Several theoretical studies have rationalized the Franck–Condon excited states of small A_n_●T_n_ duplexes (*n* ≤ 10) and showed the existence of Frenkel excitons delocalized over a few bases [35,36,37,38,39,40,41]. Due to conformational disorder, modelled via molecular dynamics simulations, the steady-state absorption spectrum of these systems was found to be an envelope of a huge number of electronic transitions, each one characterized by its own oscillator strength and polarization, as exemplified in Figure 4 of reference [42]. The most striking feature of the experimental absorption spectra is the important and wavelength-dependent hypochromism (Figure 7 and reference [42]). This effect, encountered in general for stacked DNA bases, is due to orbital overlap, generating an electronic coupling between ππ* and CT transitions [43,44,45,46,47]. For these reasons, it is not surprising that different excitation wavelengths lead to slightly different fluorescence properties (Figure 4), as reported also for pGC●pGC [22,24].

Various theoretical studies of the excited state relaxation in A_n_●T_n_ duplexes described the trapping of the Frenkel excitons by CT states [20,48,49]. According to transient absorption measurements, CT states in such oligomeric double-stranded structures are formed in high yields [6,8,50]. Depending on the excitation and probing wavelengths, the lifetimes of these states vary between 70 ps [50] and 210 ps [8]. Pure CT states, in which an entire atomic charge is transferred from one base to another, are devoid of oscillator strength and do not fluoresce at all. Thus, it is understandable that a major part of the duplex fluorescence stems from states with strong ππ* character. As a matter of fact, previous TRFS measurements on pA●pT revealed that at 330 nm, close to the maximum of the steady-state fluorescence spectrum (327 nm), 94% of the photons are emitted before 10 ps [29]. A minor longer-lived emission, detected in the visible part of pA●pT [29], had been attributed to weak coupling of CT states with bright ππ* states.

The fact that transient absorption measurements on A_n_●T_n_ have not detected any species decaying on the ns timescale [6,8,17,50] shows that the long-lived emitting states represent less than the 1% of the total excited state population. Yet, judging from the values in Figure 2 and Table 1 and Table 2, the photons emitted after 1 ns in the UV represent an important fraction of the total number of emitted photons, corresponding to ϕ values of ~3 × 10^−4^ [30]. Consequently, we conclude that true ϕ of the high energy long-lived emitters is significantly higher than those determined for monomeric constituents of the studied duplexes, (0.7 × 10^−4^ for dAMP and 1.5 × 10^−4^ for TMP) [51].

The dependence of the high-energy long-lived fluorescence, in terms of amplitude and lifetime, on the duplex size (Figure 5a) and the ionic strength (Figure 6a), shows that it is affected by the local arrangement of the bases. Similar effects were observed for the HELM excitons of the alternating duplexes (AT)_n_●(AT)_n_ [25] and (GC)_n_●(GC)_n_ [22,23]. As mentioned above, an increase in the duplex size induces an increase in the stacking distance affecting the electronic coupling between ππ* and CT transitions, which is attested by the degree of the hypochromism exhibited by the absorption spectrum, which is larger for pA●pT compared to A_20_●T_20_ (Figure 7b). It also decreases for pA●pT when the ionic strength of the buffer is lowered (Figure 6b). A correlation between the duplex size, the degree of hypochromism and the fluorescence properties has been reported for a series of double-stranded structures with alternating adenine-thymine sequences [25].

An X-ray diffraction study in solution demonstrated that the amplitude of conformational motions in adenine-thymine double-stranded structures is reduced when their size increases [52]. This is also mirrored in the vibrational coupling and the energy relaxation in double-stranded DNA, as discovered by infra-red spectroscopy [53]. Such modifications affect not only the orbital overlap between the bases, but also the dipolar coupling between the bright states of the bases, reshaping the entire network of the collective excited states. Evidently, these changes have repercussions on the relaxation pathways and, consequently, on the fluorescence properties. Thus, the fluorescence of ππ* states was found to decay faster for A_20_●T_20_ (average lifetime: 1.3 ps) than for pA●pT (average lifetime: 2.1 ps) [30].

Coming to the fluorescence anisotropy *r*, we note that the initial values found for the mononucleotides dAMP and TMP are 0.23 at 0.36, respectively [51]. However, an ultrafast depolarization of the ππ* fluorescence was observed for the examined duplexes, reaching 0.18 at the end of its decay. This was interpreted as due to energy transfer between the bases, possible only via exciton states [27,30]. The further decrease in *r*, observed in Figure 8a,c, does not arise from rotational diffusion because of the large size of the systems. In contrast, it can be correlated with a change in the polarization of the electronic transitions involved in the fluorescence. A lower limiting value for the fluorescence anisotropy of ππ* states (localized or excitonic) in B-form duplexes is 0.1; it corresponds to emission transition dipoles located within the aromatic plane of the bases that are randomly distributed in respect to the absorption while remaining orthogonal to the helix axis [54]. Anisotropies lower than 0.1 indicate the existence of out-of-plane components, e.g., coupling with CT transitions involving stacked bases. This is precisely what is observed for the long-lived fluorescence at 305 nm for both the oligomer and the polymer (*r* = 0.02, *r* = 0.04; Figure 8b,d).

Moreover, when the absorption and the emission transition dipoles are perpendicular, *r* is equal to −0.2. With this in mind, it is worth-noticing that the A_20_●T_20_ anisotropy at 420 nm attains slightly negative values on the sub-ns timescale (−0.01 in Figure 8c), revealing emission from excited states with stronger CT character. However, *r* eventually increases and becomes slightly positive on the ns timescale (Figure 8d). This means that the excited states with weaker CT components have longer lifetimes than those with stronger CT.

We remark that the gap between the *r* traces determined for pA●pT at 305 and 420 nm is smaller than that observed for A_20_●T_20_ (Figure 8). A similar trend was reported for the fluorescence decays and anisotropies determined for the two duplexes on the ps timescale by fluorescence upconversion, those of the oligomer exhibiting a larger dispersion with the emission wavelength [30]. This is also reflected in their steady-state fluorescence spectra (Figure 1), whose width at half maximum is larger for the oligomer (6900 cm^−1^) than that for the polymer (5400 cm^−1^). We explain these observations by the fact that the more efficient base stacking in the polymeric duplex induces a faster mixing between ππ* and CT states, while imposing constraints for the geometrical rearrangement required for excited states with strong CT character. It is worth stressing that, despite these differences, the overall ϕ of the two systems is the same within 15%.

## 4. Materials and Methods

### 4.1. Materials and DNA Handling

pA●pT was obtained as a duplex by Amersham Biosciences. DNA single strands A_20_ and T_20_, purified by reversed phase HPLC and tested by MALDI-TOF, were purchased from Eurogentec. For the formation of A_20_●T_20_, a thermal treatment in a dry bath (Eppendorf-ThermoStatplus) was applied to an equimolar mother solution of the constitutive single strands (2 mL; oligomer concentration: ~10**^−^**^3^ mol●L**^−^**^1^): the solution, after being heated at 96 °C, was cooled to the melting point of the system (cooling time: 1 h), where the temperature was maintained for 10 min; subsequently, the solution was cooled to 4 °C (cooling time: 2 h), where it was incubated overnight. Ultrapure water was delivered by a MILLIPORE (Milli-Q Integral) system. Melting curves were obtained with a Perkin-Elmer Lambda 900 spectrophotometer. The temperature of the solution was controlled by a Huber CC3 apparatus with a precision of ±0.1 °C.

### 4.2. Time-Resolved Measurements

The laser source was a commercial mode-locked Ti:Sapphire (MIRA 900, Coherent), pumped by a cw solid state laser (VERDI V10, Coherent), delivering ~120 fs pulses at 795 nm with about 2 W average power at 76 MHz repetition rate. The infrared laser beam was used to pump an optical parametric oscillator (MIRA-OPO, Coherent), delivering visible ~120 fs pulses with an average power of ~0.5 W. The 267 and 285 nm excitation beams were generated by focusing the visible laser beam into a 5 mm BBO type I crystal. The repetition rate was reduced at 4.75 MHz by using a pulse picker (Model 9200, Coherent). The fluorescence from the sample cell, filtered by a Schott WG 295 filter, was collected and imaged by two parabolic mirrors on the input slit of a small monochromator (Jobin-Yvon HR25). The detector was a microchannel plate (R1564 U, Hamamatsu) providing an instrumental response function of ~80 ps (FWHM), as given by the Raman line of water. The TCSPC setup used a SPC630 card (Becker & Hickl).

Fluorescence decays were recorded at the magic angle (54.7°). For the determination of fluorescence anisotropy *r =* (*I_par_* − *I_perp_*)/(*I_par_* + *2I_perp_)*, parallel (*I_par_*) and perpendicular (*I_perp_*) components of the fluorescence decay were recorded by controlling the polarization of the exciting beam with a half-wave plate. The excitation energies under parallel and perpendicular conditions were identical, giving a G factor of 1.

To avoid photodegradation, mainly due to thymine dimerization [55] and photoionization [56], specific experimental protocols, described in detail in reference [57] were followed. Briefly, 3 mL of the DNA sample, contained in a 1 × 1 cm^2^ quartz cell, were continuously stirred. The exciting beam was defocused on the surface decreasing its intensity to 3 kW●cm**^−^**^2^. Moreover, fresh samples were used to record signals at each emission wavelength. We systematically checked that the successive measurements provided identical decays; the data sets were finally merged to increase the signal-to-noise ratio.

## 5. Conclusions and Perspectives

Our present time-resolved fluorescence study on the DNA duplexes pA●pT and A_20_●T_20_ unveiled the existence of high-energy long-lived fluorescence stemming from mixed ππ*/CT states, which is not detectable in their steady-state emission spectra. Associated to previous studies performed for the same systems on a shorter timescale [1,27,29,30], they demonstrate the important role played in the excited state relaxation by the size of the duplex. It would be interesting to explore this effect by transient absorption measurements which detect dark CT states, going undetected by fluorescence measurements.

**Figure 9:**
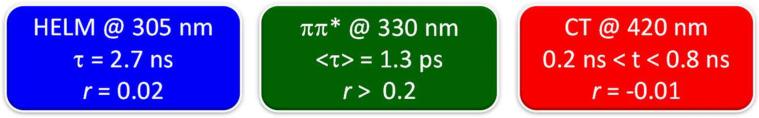
Limiting types of emitting states detected for A_20_●T_20_; combined data from this work and reference [30].

In Figure 9, we summarize the three limiting types of excited states whose emission is dominating over various probed time-domains. This is depicted in the case of the oligomeric duplex, for which transient absorption data regarding the CT states are also available. Below 10 ps, we have emission from ππ* states, whose average lifetime is 1.3 ps. Then, these states start acquiring an increasing CT character. Excited states with strong CT character emit between 0.2 and 0.8 ns, in line with the findings of transient absorption spectroscopy [6,8,50]. Finally, the longest-lived emission detected here (2.7 ns) is characterized by a smaller CT character and its major part appears in the blue side of the spectrum.

The behavior of the high-energy long-lived excited states of pA●pT and A_20_●T_20_, in terms of fluorescence lifetime and anisotropy, as well as their dependence on the duplex size and the ionic strength, strongly resembles that reported for the HELM excitons of (GC)_n_●(GC)_n_ and (AT)_n_●(pAT)_n_ duplexes. As mentioned in the introduction, quantum chemistry calculations on such alternating duplexes assigned the high-energy long-lived excited states to mixed excitons. They were found to extend over at least four bases and be characterized by a weak (q < 0.2 au) intrastrand transfer (G^+δ^ → C^−δ^ or A^+δ^ → T^−δ^) [24,25]. These excitons were correlated with specific coplanar arrangements of base pairs, which can be easily destroyed by conformational motions. Evidently, such a picture is not compatible with duplexes containing solely adenine and thymine tracts. Specific theoretical studies on homopolymeric systems are needed to identify the type bases (adenines, thymines or both) involved in the charge transfer and those participating to exciton coupling. To catch the strong dependence of the long-lived emitting states on conformational factors, a combination of molecular dynamics simulations with quantum chemistry methods is indispensable. This is an important challenge, which may be proved worthy, as high-energy long-lived excited states could lead to photochemical reactions damaging the genetic code.

## Figures and Tables

**Table 1:** Comparison of the % of photons emitted after 1 ns at 305 and 330 nm. Total time-range considered: −1–10.5 ns. Excitation wavelength: 285 nm.

Emission Wavelength	pA●pT (Standard Buffer)	A_20_●T_20_ (Standard Buffer)	pA●pT (Sixfold Diluted Buffer)
305 nm	27.5%	50.1%	31.7%
330 nm	12.5%	30.6%	15.7%

**Table 2:** Relative importance *R* of the photons emitted by the studied duplexes after 1 ns at 305 in respect to those emitted at 420 nm; the corresponding time constants ^1^ are shown in brackets.

Excitation	pA●pT	A_20_●T_20_
267 nm	5.6 (2.6 ns)	-
285 nm	3.6 (2.2 ns)	3.7 (2.7 ns) ^1^

^1^ Derived from fits on the 1.5–10.5 ns range.

## Data Availability

Not applicable.

## Supplementary Materials

The following supporting information can be downloaded at: https://www.mdpi.com/article/10.3390/molecules27113558/s1, Figure S1: Time-resolved fluorescence spectra; Figure S2: Fits of the fluorescence decays.

## Sample Availability

DNA samples and other chemicals are commercially available.
