# Supplementary material for: High-Energy Long-Lived Emitting Mixed Excitons in Homopolymeric Adenine-Thymine DNA Duplexes
Source: Molecules. 2022 May 31;27(11):3558. doi: 10.3390/molecules27113558 (PMC9181881; doi:10.3390/molecules27113558)
Supplement: Supplementary file 1 [file molecules-27-03558-s001.zip › molecules-1732012-SI.pdf]

High-energy long-lived emitting mixed excitons in homopolymeric adenine-thymine DNA duplexes

Ignacio Vayá [1,2], Thomas Gustavsson[2] and Dimitra Markovitsi [2,3*]

[1] Departamento de Química, Instituto de Tecnología Química UPV-CSIC, Universitat Politècnica de València, 46022, Valencia, Spain;
igvapre@qim.upv.es (I.V.)
[2] Université Paris-Saclay, CEA, CNRS, LIDYL, F-91191 Gif-sur-Yvette, France;
thomas.gustavsson@cea.fr (T.G.)
[3] Université Paris-Saclay, CNRS, Institut de Chimie Physique, UMR8000, 91405 Orsay, France
dimitra.markovitsi@universite-paris-sacaly.fr (D.M.)
* Correspondence: dimitra.markovitsi@universite-paris-saclay.fr

SUPPORTING INFORMATION

Figure S1: Time-resolved fluorescence spectra

Figure S2: Fits of the fluorescence decays

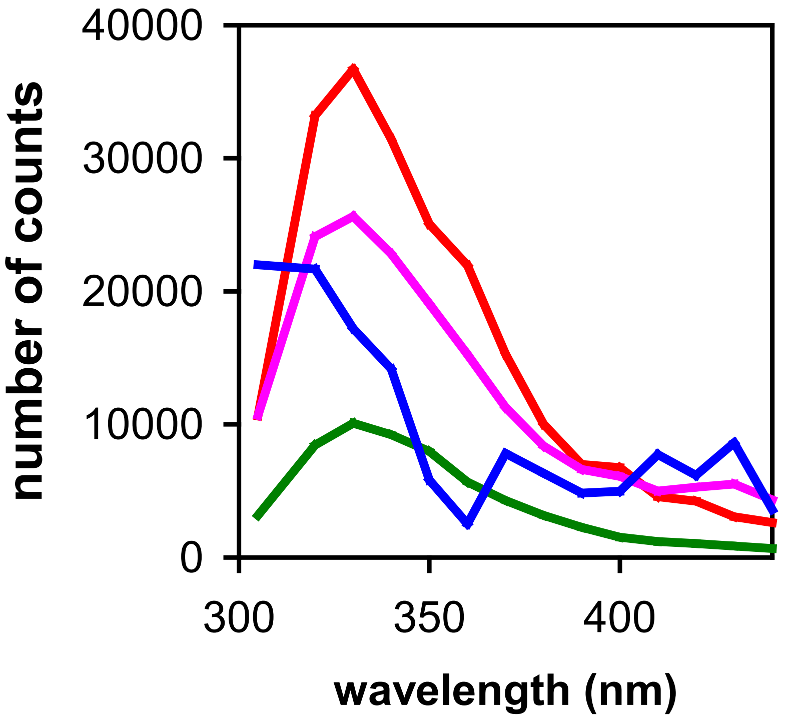

Figure S1. Time-resolved fluorescence spectra, non-corrected for the response of the detection system, determined for pA•pT by integrating the photons emitted on four time-windows: -10 -10 ps (green), 10 – 100 ps (pink), 0.1 -1 ns (red), and 1 - 10 ns (blue).

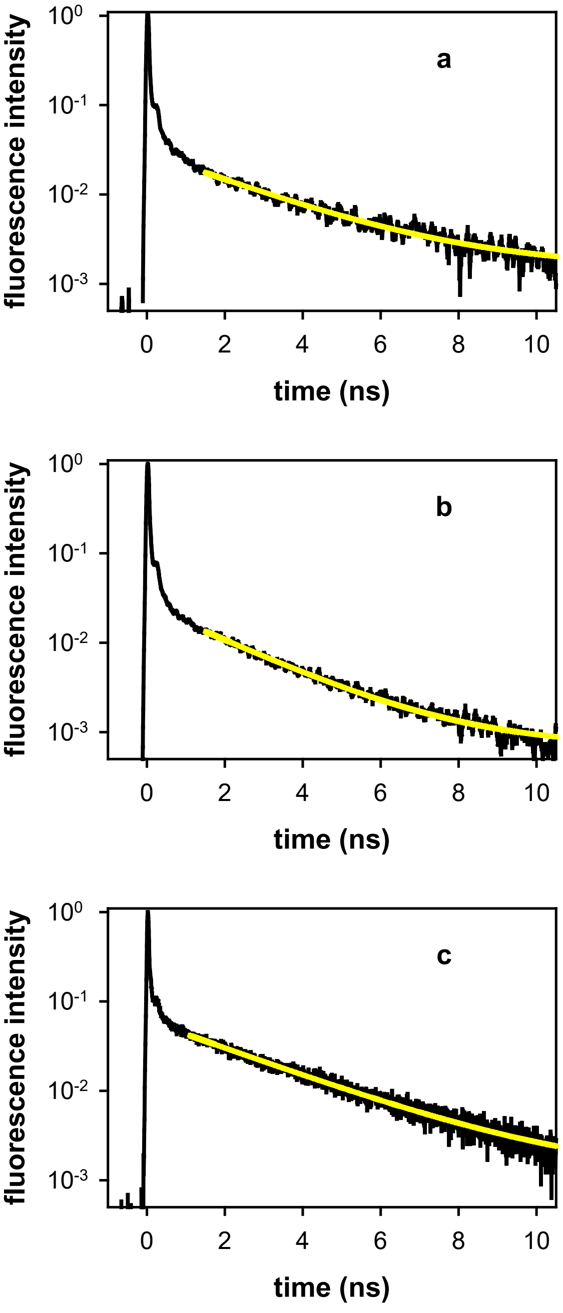

Figure S2. Fits of the fluorescence decays recorded at 305 nm with mono-exponential functions performed on the 1.5 – 10.5 ns range. (a) pA•pT, excitation wavelength: 267 nm; (b) pA•pT, excitation wavelength 285 nm; (b) A_{20}•T_{20}, excitation wavelength: 285 nm.
